# Effect of Morphological Changes due to Increasing Carbon Nanoparticles Content on the Quasi-Static Mechanical Response of Epoxy Resin

**DOI:** 10.3390/polym10101106

**Published:** 2018-10-06

**Authors:** Hamed Yazdani Nezhad, Vijay Kumar Thakur

**Affiliations:** Enhanced Composites & Structures Centre, School of Aerospace, Transport & Manufacturing, Cranfield University, Milton Keynes MK9 2BS, UK

**Keywords:** polymers, composites, carbon nanoparticles

## Abstract

Mechanical failure in epoxy polymer and composites leads them to commonly be referred to as inherently brittle due to the presence of polymerization-induced microcrack and microvoids, which are barriers to high-performance applications, e.g., in aerospace structures. Numerous studies have been carried out on epoxy’s strengthening and toughening via nanomaterial reinforcement, e.g., using rubber nanoparticles in the epoxy matrix of new composite aircraft. However, extremely cautious process and functionalization steps must be taken in order to achieve high-quality dispersion and bonding, the development of which is not keeping pace with large structures applications. In this article, we report our studies on the mechanical performance of an epoxy polymer reinforced with graphite carbon nanoparticles (CNPs), and the possible effects arising from a straightforward, rapid stir-mixing technique. The CNPs were embedded in a low viscosity epoxy resin, with the CNP weight percentage (wt %) being varied between 1% and 5%. Simplified stirring embedment was selected in the interests of industrial process facilitation, and functionalization was avoided to reduce the number of parameters involved in the study. Embedment conditions and timing were held constant for all wt %. The CNP filled epoxy resin was then injected into an aluminum mold and cured under vacuum conditions at 80 °C for 12 h. A series of test specimens were then extracted from the mold, and tested under uniaxial quasi-static tension, compression, and nanoindentation. Elementary mechanical properties including failure strain, hardness, strength, and modulus were measured. The mechanical performance was improved by the incorporation of 1 and 2 wt % of CNP but was degraded by 5 wt % CNP, mainly attributed to the morphological change, including re-agglomeration, with the increasing CNP wt %. This change strongly correlated with the mechanical response in the presence of CNP, and was the major governing mechanism leading to both mechanical improvement and degradation.

## 1. Introduction

Polymer-based particulate nanocomposites prepared using particulates with small aspect ratios represent one of the emerging classes of composite materials [1,2,3,4,5,6,7,8,9,10]. Mechanical properties of nanocomposite materials such as toughness, stiffness, and strength are significantly affected by the interface adhesion amongst the particle and polymer matrix, size/shape of the particle, and its loading [11,12,13,14,15]. These properties are of priority to the main structural analysis [16,17,18]. Different techniques and manufacturing processes for particulate polymer composites are reviewed in several publications and reports, such as a comprehensive study for calcium carbonate (CaCO_3_) in [9] and for a number of 2D and 3D layered nanostructures in [19,20] or for dielectric heating purposes [21]. Enhancements in different mechanical properties have been addressed in [9] with the addition of a few weight percentage (wt %) of carbon nanomaterials, e.g., carbon nanotubes, because in thermosetting resins inorganic particles were found to be imperative fillers/reinforcement. It is well-known that with the incorporation of micro/nano-particles, Young’s modulus can be enhanced because the rigid inorganic particles commonly exhibit higher stiffness in comparison to the polymer matrices. However, the quality of the bond between nanoparticle and polymer has been proven to play a significant part in the structural strength [9], i.e., interface adhesion among the reinforcing particle–polymer matrix and content of the particle (i.e., loading) are the two most imperative aspects affecting the mechanical properties.

However, a substantial reduction in the fracture toughness of thermoplastic polymers reinforced with rigid particulates in comparison to the as-received polymers has been reported [22]. On the other hand, some studies have also reported on the increment in toughness of thermoplastics such as polypropylene/polyethylene with the incorporation of rigid particles in [4,6]. An increase in impact toughness has also been described in the composites of polyethylene reinforced with CaCO_3_ particles [4,6,8]. According to [4], nanoparticle incorporation into the polymers results in greater rigidity as well as better yield strength. It has been reported that reinforcement of polypropylene with calcium carbonate particles imparts good strength to the resulting composites at a particular loading. It has also been shown by several authors that the modulus is not affected to a large degree in studies where the mean particle diameter was greater than 100 nm [3,9,23]. These studies were focused on silica/epoxy, aluminum hydroxide filled polypropylene, CaCO_3_-filled polybenzoxazine, aluminum particle/polyester, and organo-soluble polyimide/silica material systems. Conversely, when the particle size is reduced below 100 nm, there is a clear correlation between particle size and the modulus. Polypropylene/CaCO_3_ composites containing smaller nanoparticles (21 nm) were shown to exhibit a higher Young’s modulus in comparison to the nanoparticles with larger size (39 nm) [2], having characterized the nanoparticles using X-ray diffraction. Thus, it’s been suggested that above a critical particle size, no further degrading effect on the modulus of the prepared composites takes place. Furthermore, the magnitude of the critical particle size has been found to be dependent on the type of particle/reinforcement polymer matrix as well as particle/polymer matrix adhesion [24]. In contrast to the effective particle size, treatment for the purpose of increasing the particle-polymer interfacial adhesion exhibits a lesser or negligible effect on the mechanical stiffness. For particulate reinforcement/particle loading (i.e., volume fraction) the composite strength has been found to increase with a decrease in particle size [25], as smaller particles increase the total interfacial surface area. In an interesting work, the effect of the size of the reinforcing particle on the strength of PA6/silica nanocomposites with mean sizes of 12, 25, and 50 nm in [26] was studied and it has been shown that with the incorporation of particles, an increase in strength is observed. In fact, the adhesion quality between the polymer matrix and the reinforcement affects the overall composite strength and toughness. The toughness is also affected by the size of the particle, interfacial adhesion, and particle content level. It is expected that in a ductile medium (e.g., thermoplastics), the inclusion of fillers increases the brittleness of composite if there is limited/no interfacial adhesion. In case of polymer matrices having brittle characteristics (e.g., thermoset epoxy), a reverse phenomenon is expected to occur as the particles introduce a crack arrest mechanism to the brittle medium, thus considerably reducing the brittleness. Moreover, micro-cavitation and micro-debonding can easily occur in such a medium during composite manufacturing or mechanical loading and cracks can coalesce.

Generally speaking, the bond equality is often attributed to process control parameters such as ultrasonication, dispersion, surface functionalization, treatment and mixing techniques. Control over numerous parameters may become an efficiency challenge (in terms of adding extra time and cost) for industrial applications, and as such, industries tend to follow straightforward mixing processes. Also, design against brittle failure (the case of epoxy polymers; this study) in nanocomposites is of critical importance. In a given bulk polymer material, the transition from a brittle to a ductile fracture is significant as the fracture behavior is considerably affected by the internal/external conditions. Thermoset epoxies are intensely used in structural applications despite the fact their brittleness has necessitated the incorporation of inclusions, e.g., particulate fillers. In the toughness study of nano-filled polymers, plane strain and plane stress driven cracks are superimposed to determine the impact of the incorporation on the modification of the polymer’s bulk properties, meaning that if, in the nano-filled polymer, regions with plane stress driven fracture are provoked within the space between nanoparticles, the polymer will tend to yield longer. Thereby, a macroscopic brittle matrix filled by nanoparticles may display localized plastic deformation [27,28,29]. Accordingly, incorporation of carbon nanoparticles in polymers reduces the polymer resistance to plastic deformation by allowing cavitation and debonding prior to plastic flow. Thus, for a carbon nanoparticle (CNP) filled epoxy, the strength depends on the effective stress transfer among the reinforcing particle and polymer. This is driven by adhesion and therefore geometry-related morphological features can become highly influential. On the other hand, nano-composite stiffness depends on the particle content, not the adhesion. This is attributed to a significantly greater modulus of the particles than the polymer matrix [30]. In this paper, the mechanical response of the bulk of CNP filled epoxy has been studied by focusing on the effects of morphological variation with increasing CNP content. The epoxy has been chosen as the matrix because of its superior mechanical properties; resistance to stresses (e.g., thermal), low viscosity which facilitates the dispersion of reinforcement; low shrinkage, improved adhesion to the targeted substrate, and degradation over time to name only a few [14,17,29]. The study has been performed in two micro- and macro-scales: Bulk and CNP reinforced specimens were manufactured and exposed to tension, compression, and nanoindentation. As the CNP loading content plays a significant role in the determination of the bulk response, mechanical properties (modulus, hardness, strength, and failure strain) were studied with the variation of CNP wt %.

## 2. Materials and Methods

### 2.1. Epoxy Resin

The epoxy matrix composite was prepared by first mixing a low viscosity epoxy precursor (Epoxy equivalent weight 117–133 g/eq), with Methyl nadic anhydride (MNA) that acts as a hardener with 2-Dodecenyl succinic anhydride (DDSA). Since the viscosity of the various ingredients differs, it is imperative to mix all the ingredients thoroughly. The mix bundle was stirred for a few hours before adding the curing agent. The epoxy precursor, hardeners, and curing agent were supplied by Sigma-Aldrich, Dorset, UK. 2,4,6-Tri (dimethyl aminomethyl) phenol (DMP-30) was used as the curing agent in this work. According to the epoxy specifications, the viscosity reached 60 cps which is approximately 10 % of the viscosity of conventional resins for composite applications and thus facilitated the CNP incorporation.

The final epoxy mixture was gently injected to molds suitable for the test stages (tensile, compression, and nano-indentation) and was cured at 80 °C for 12 h in a conventional vacuum-assisted heating oven. Higher temperature curing was not used to avoid any possible drawback on the CNP-epoxy interfaces, e.g., the interfacial pre-existing dis-bond due to thermally-induced residual stresses. As per our differential scanning calorimetry (DSC) investigation, the *T*_g_ (glass transition temperature) for the final mixture was 77 °C.

### 2.2. Release Agent

The high-performance solvent-free polymer release agent, Zyvax® Watershield, was applied to the mold surfaces three times for easy mold release. Each time the agent was allowed to dry out on the surfaces for 30 min. The mold was then closed, and the final mixture was injected.

### 2.3. Curing Agent

A proportion of 1.5–2.0% of DPM-30 was added to the mixture shortly after CNPs were added and stirred for 12 h at room temperature (RT). The 1.5–2% DPM-30 enhances the strength and adds brittleness to the final mixture according to the supplier’s specifications. The temperature of the mixture was not increased before adding the curing agent in order to ensure a slower rate of cross-linking. The final mixture with the CNPs was then stirred under vacuum conditions using a Schlenk line at RT with a magnetic stirrer. The mixture was de-gassed after adding the curing agent, at <5 kPa (0.05 atmospheres).

### 2.4. Adding Nanoparticle to the Epoxy Mixture

Simple short-step processing methods are highly important for industrial applications as they benefit mass production. A simple mixing method is suggested herein: The epoxy mixture was heated up to 100 °C before the CNPs were added. Heating provides a mixture with a relatively low viscosity that leads to a good CNP dispersion [22]. CNPs, supplied by Sigma-Aldrich with an average size of 50–100 nm (molecular weight of 12.01 g/mol), were then directly added to the mixture, according to the direct addition procedure in [31]. The mixture of the nanoparticles and the epoxy/curing agent bundle was stirred under vacuum conditions, and then allowed to cure after injecting into the mold under 1 kPa (<0.01 atmosphere) vacuum conditions in a vacuum-assisted oven. It has also been shown that the CNP clusters can easily disperse in a low polar epoxy matrix, resulting in a stable dispersion [31]. According to [20,32], one of the current approaches for exfoliation is the exposure of a layered material ultrasonic waves. However, it has further been discussed in [20] that the ultrasonication process (in surfactant/polymer solutions) contributes to the electrostatically/sterically stabilized graphene nanosheets in a solvent. In our heated low viscosity epoxy resin, a well dispersed CNP was expected. Our testing data, to be presented in the following sections, also exhibit consistent trends obtained from nanoindentation and macro-scale mechanical testing, which represent well-dispersed CNPs in the epoxy. Note that re-agglomeration post-stirring was observed; however, due to the comparative nature of this study and the focus on morphological features, it was considered as a constant parameter for all samples.

Based on preliminary trials, the following 30-h procedure (eight stages), detailed in Table 1, was used for the development of a well-dispersed less-agglomerated CNP filled epoxy. The time between stages 4 and 5 can be reduced depending on how long it takes to achieve a satisfactory viscosity for injection purposes.

### 2.5. Injection Molding of CNP Filled Epoxy

A mixture of approximately 30 mL epoxy was injected into an aluminum mold. The mold was cleaned using acetone, and dried using compressed air. A Zyvax® liquid sealer was first applied to protect the mold from micro-porosity, followed by application of the Watershield release agent onto the mold surfaces. Watershield was applied two or three times and was allowed to dry each time for 30 min. The CNP-epoxy mixture was then injected.

### 2.6. Test Specimens

**Figure 1:**
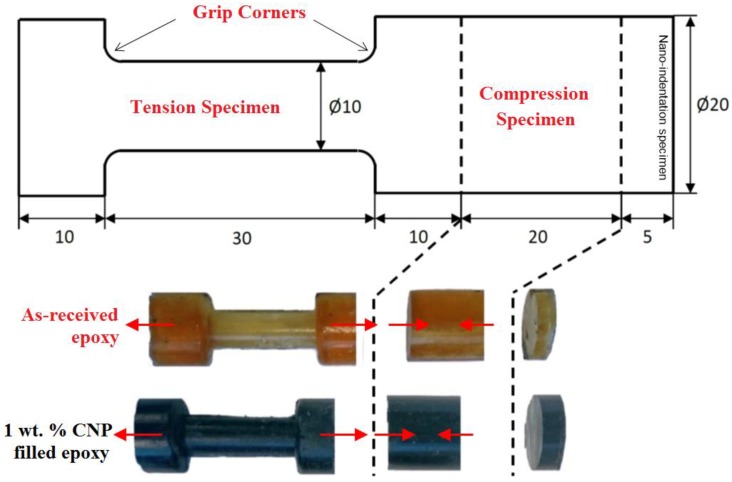
Cylindrical tension, compression and Nanoindentation test specimens extracted from the mold (all dimensions in mm; arrows show loading direction) from CNP filled epoxy composite.

Three sets of test specimens were extracted from the mold as shown in Figure 1. Tensile specimens (Ø10 mm), compressive specimens (Ø20 mm), leftover material for nanoindentation and SEM investigation. Figure 1 shows the 1 wt % CNP filled epoxy and as-received specimens extracted from the injection mold. Arrows show the direction of uniaxial loads applied using a Tinius-Olsen benchtop machine (Tinius-Olsen, Salfords, UK).

## 3. Results and Discussion

Initial investigations on the morphology and dispersion of the CNPs in cured specimens were carried out before the mechanical testing.

### 3.1. Morphology and Composition

**Figure 2:**
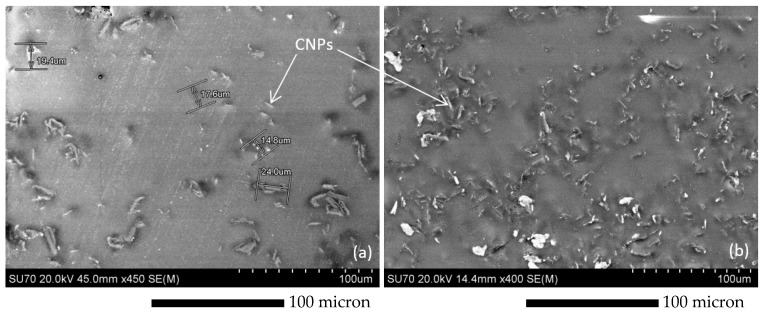
SEM images of (**a**) 2 wt % and (**b**) 5 wt % CNP filled epoxy specimens, showing relatively long rod shape nanoclusters in 2 wt % specimens (~20 micron long) as opposed to relatively short and agglomerated nanoclusters in 5 wt % specimens (~10 micron long).

A typical site of 2 and 5 wt % CNP filled epoxy specimens is presented in Figure 2. Both samples were gold coated (20 mA, one-round coating) to facilitate electron discharge from the surface of the specimen. The CNPs are observed, mostly, as rod shape fillers dispersed in epoxy, with sizes ranging from 5 to 30 microns, and the two specimens differ in terms of dispersion density.

**Figure 3:**
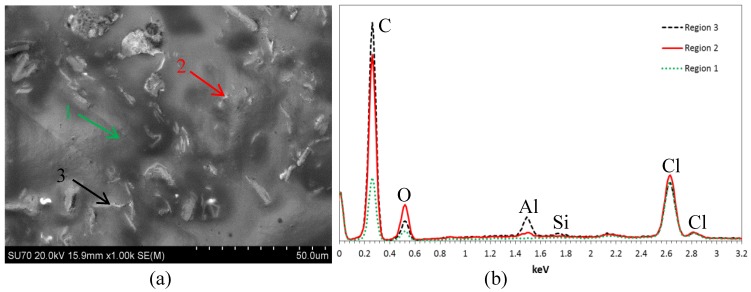
EDS data from 5 wt % CNP filled epoxy, depicting correct identification of rod shape CNP nanoclusters at Regions 2 and 3; (**a**) SEM image, (**b**) EDS data.

To ensure the presence of the CNPs observed in the SEM images, a quick survey of the composition was carried out in-situ using the energy dispersive X-ray spectroscopy (EDS) instrument in conjunction with the SEM. EDS data is shown in Figure 3 from three regions of the 5 wt % CNP filled epoxy: Region 1 over an area with no rods, and Regions 2 and 3 over two randomly picked rods. EDS data clearly show the presence of chlorine and Si at the same content level for all regions. The presence of Al is due to the use of the polishing suspension during the preparation of the specimens for SEM. The EDS data from Regions 2 and 3, i.e., rods, show a higher content of carbon and oxygen compared to Region 1. This implies that the observed rods are made of CNP.

### 3.2. 3D Dispersion

**Figure 4:**
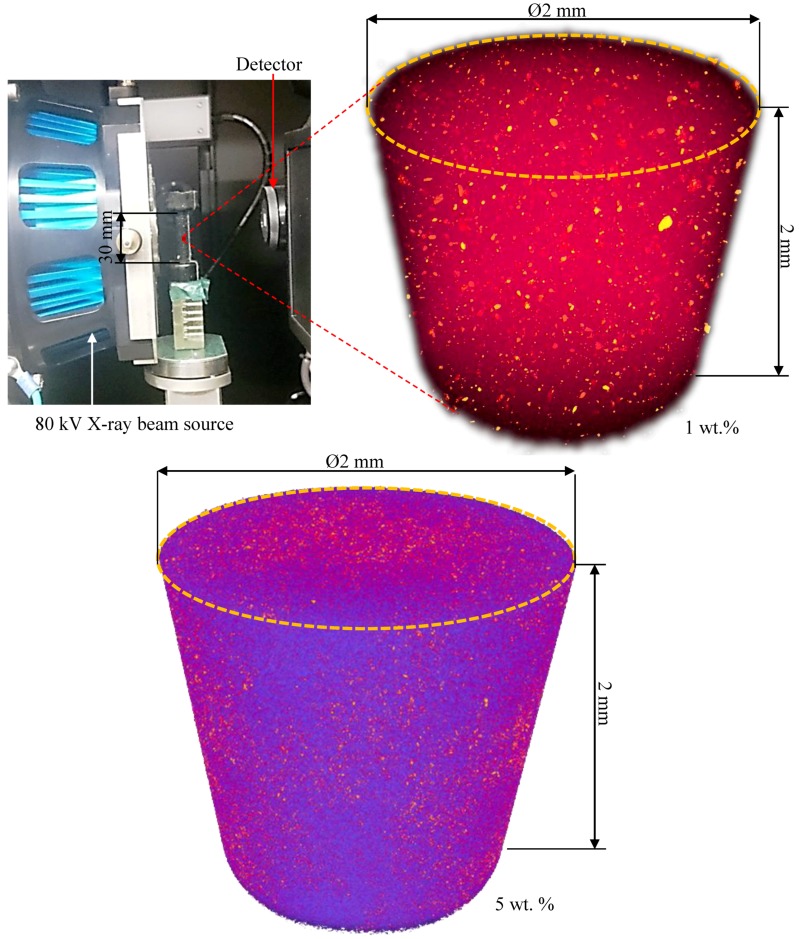
3D X-ray computed tomography of 1 and 5 wt % CNP embedded epoxy (yellow spots represent CNPs as nanoclusters) at the center of test specimens covering a cylindrical region of Ø2 mm × 2 mm; results for 1 wt % specimens show large discrepancies in the size of CNPs while small discrepancies for 5 wt % specimen.

The SEM images presented a CNP dispersion with a 2D architecture. Using the phase contrast imaging capabilities of the advanced computed tomography system, VersaXRM-500 (Xradia, CA, USA) using a high-energy X-ray source (80 kV), high-resolution 3D images of the internal morphology of the nanoparticle embedded epoxy were captured. Impurities such as nanoparticles are observed in yellow (bright spots in black and white), as shown in Figure 4, which show dispersion in 1 wt % CNP embedded epoxy at the center of the specimen, a cylindrical region of Ø2 mm × 2 mm. Voids and air traps appear as dark spots. This indicates a well-dispersed CNP-epoxy structure, achieved through the vacuum-assisted embedment procedure described in Table 1. The primary investigations show that the maximum CNP cluster size is varied between 1–60 microns. The image also indicates a distinct lack of significant air pockets post-cure, a sign of the efficient use of vacuum-assisted injection. Moreover, they show large discrepancies in the size of CNPs for 1 wt % specimens, while small discrepancies for 5 wt % specimen are shown.

### 3.3. Mechanical Testing

#### 3.3.1. Nanoindentation

**Figure 5:**
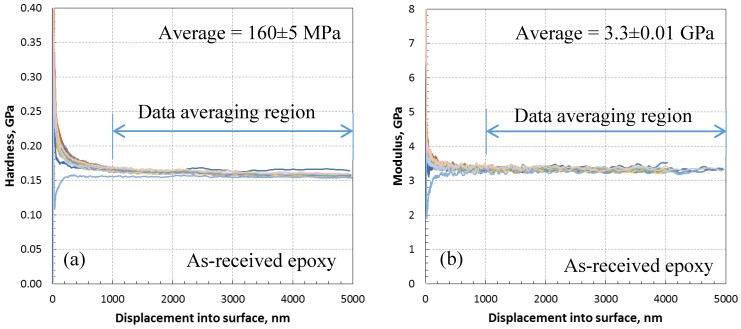
Nanoindentation data of as-received (0 wt % CNP) epoxy; (**a**) variation of hardness with depth showing steady hardness after 1 micron indentation with average hardness of 160 ± 5 MPa, (**b**) variation of modulus with depth showing steady modulus after 1 micron indentation with an average modulus of 3.3 ± 0.01 GPa.

The nanoindentation experiments were performed using a Nano Indenter G200 supplied by Agilent Technologies (Keysight Technologies, Wokingham, UK). The G200 uses a Nano-Mechanical Actuating Transducer (NMAT) to apply loads and measure displacements during nanoindentation tests. The load is controlled by electromagnetic actuation and the displacement is measured using a capacitance gauge. In order to characterize the specimens, indentation experiments were carried out on the specimens using the dynamic continuous stiffness measurement technique. This technique allows for continuous measurement of the contact stiffness through the indentation loading. A strain rate target of 0.05/s and a maximum depth of 5 µm was assigned to the indentations, and a Berkovich indenter pyramid tip was used. A total of 20 indentations were carried out randomly over the surface of each sample. Figure 5 shows the modulus and hardness results for the as-received epoxy sample. The results are reasonably consistent with depth for very low scattered data. Averaging the results from the depth of 1 to 5 µm gave mean modulus and hardness values of 3.3 ± 0.01 GPa and 160 ± 5 MPa for the as-received epoxy, respectively.

**Figure 6:**
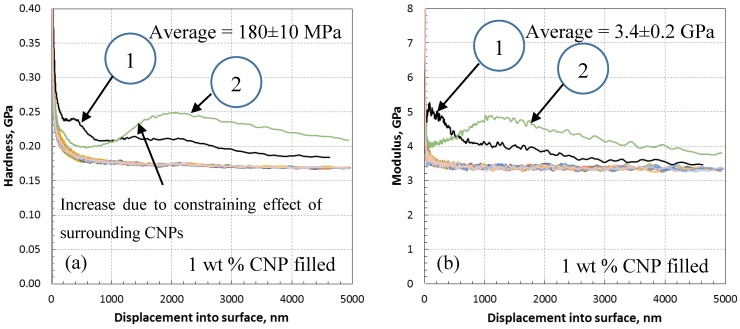
Nanoindentation data of 1 wt % CNP filled epoxy; (**a**) variation of hardness with depth showing non-steady hardness at or near CNPs (outliers 1 and 2 in Figure 7) with an average hardness of 180 ± 10 MPa, (**b**) variation of modulus with depth showing non-steady modulus at or near CNPs (outliers 1 and 2 in Figure 7) with average modulus of 3.4 ± 0.2 GPa.

**Figure 7:**
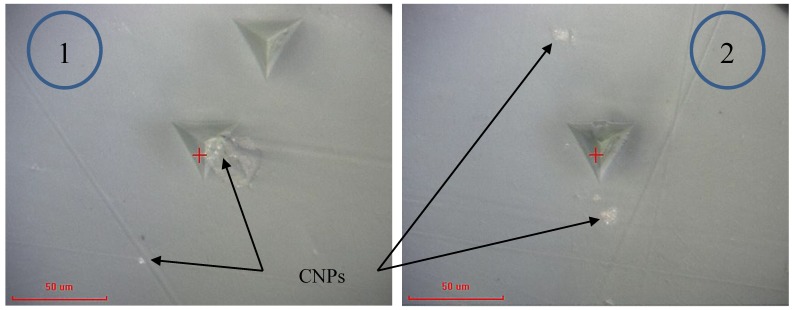
Micrographs of the indentation site of 1 wt % CNP filled epoxy for outliers 1 and 2 in Figure 6, representing indentation at or near CNP clusters.

Figure 6 presents the modulus and hardness for the epoxy embedded by 1 wt % CNP. The data for this specimen are very similar to the as-received epoxy with only a slight increase in the mean modulus (3.4 ± 0.2 GPa) and hardness (180 ± 10 MPa), a 3% and 12.5% increase, respectively. This increase is likely due to the presence of CNPs and nanoindentation targeting CNP clusters within two outliers which have been highlighted on the curves. Micrographs of the indentation sites for the outliers 1 and 2 are shown in Figure 7. It is clear that the indentation was carried out very close to what appears to be a large cluster of nanoparticles. The indentation properties for outliner 2 gradually reach the maximum levels at larger indentation depths compared to those of outliner 1, likely due to the presence of a cluster underneath the indenter (not visible in Figure 7). Note that this increase is gradual, and not rapid as it is for the case of fiber-reinforced composites where the indenter comes into contact with fibers in a densely distributed fibrous matrix (see, e.g., [33]). This is expected for the case of 1 wt % and is a sign of a low constraining effect of surrounding nanoparticles as the dispersion density shown in Figure 7 is relatively low.

**Figure 8:**
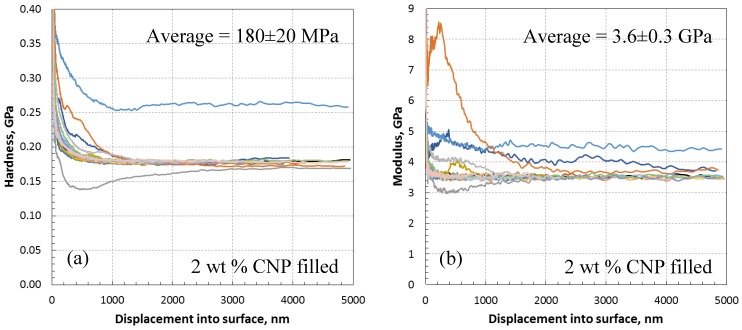
Nanoindentation data of 2 wt % CNP filled epoxy; (**a**) variation of hardness with depth showing non-steady hardness at or near CNPs with average hardness of 180 ± 20 MPa, (**b**) variation of modulus with depth showing non-steady modulus at or near CNPs (Figure 9) with average modulus of 3.6 ± 0.3 GPa.

The modulus and hardness results for 2 wt % CNP filled epoxy are presented in Figure 8, giving mean modulus and hardness values of 3.6 ± 0.3 GPa and 180 ± 20 MPa, respectively. The 2 wt % inclusion of CNP improves the hardness of the as-received epoxy by 12.5%, while the modulus is increased by 8% compared to the as-received epoxy. This is higher than the increase obtained in the 1 wt % inclusion and is thought to be due to the presence of relatively higher CNPs compared to the 1 wt % specimens.

**Figure 9:**
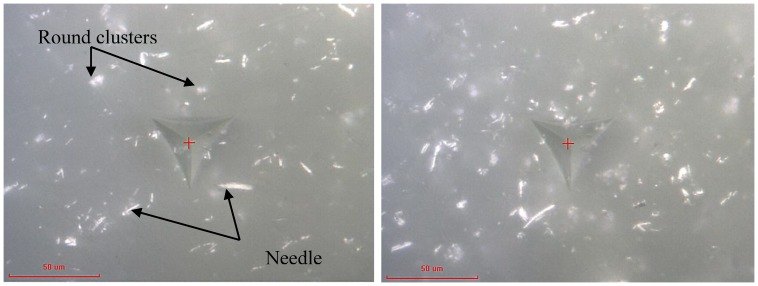
Micrographs of the indentation site of 2 wt % CNP filled epoxy, representing indentation at or near CNP clusters.

The morphology of the indentation site is provided in Figure 9. As shown, the clusters are densely distributed for this case, as opposed to the 1 wt % case shown in Figure 7, and the agglomeration and shape of the CNP clusters are also different. The figure indicates that a combination of round and rod shape CNPs occurred during the embedment and curing procedure. The rod shape CNPs were formerly observed in the SEM images of the 2 and 5 wt % specimens, see Figure 2.

**Figure 10:**
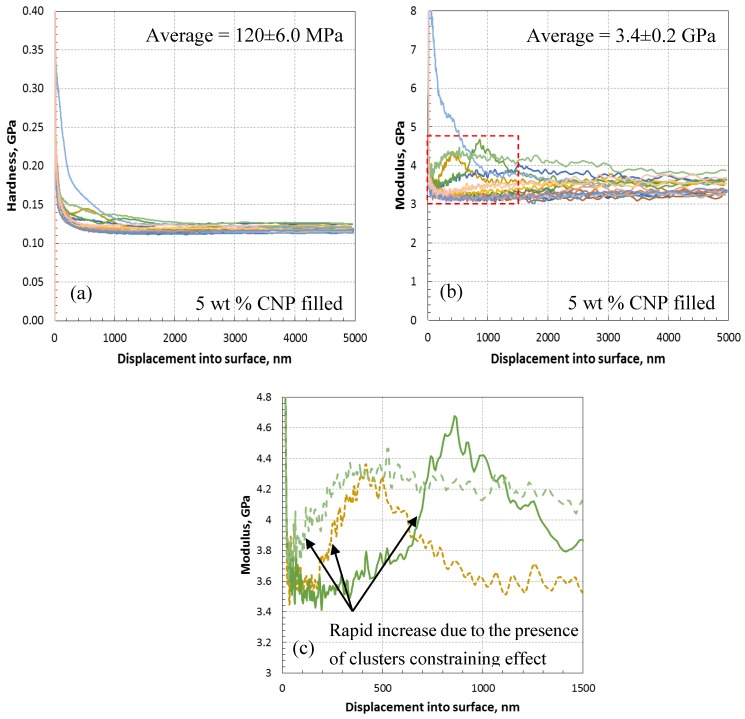
Nanoindentation data of 5 wt % CNP filled epoxy; (**a**) variation of hardness with depth showing steady hardness at or near CNPs (Figure 11) with average hardness of 120 ± 6 MPa, (**b**) variation of modulus with depth showing steady and scattered modulus at or near CNPs (Figure 11) with average modulus of 3.4 ± 0.2 GPa, (**c**) magnified view of dashed-line region from subfigure (**b**).

**Figure 11:**
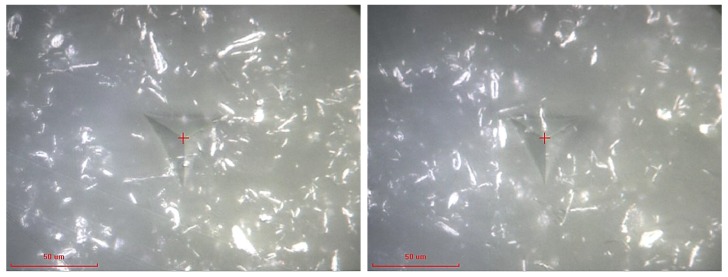
Micrographs of two indentation sites of 5 wt % CNP filled epoxy, representing indentation at or near CNP clusters.

Figure 10 presents the modulus and hardness for the epoxy incorporated by 5 wt % CNP showing highly scattered data obtained from the 20 randomly distributed nanoindentation data. Mean modulus and hardness values are 3.4 ± 0.2 GPa and 120 ± 6 MPa, respectively. Compared to the 1 and 2 wt % cases, shown in Figure 6 and Figure 8, no apparent improvement in mean modulus and an apparent reduction in mean hardness is observed. This can be attributed to the morphology and dispersion of CNP. A morphological scan of the indentation site presented in Figure 11 shows that rod-shaped CNPs are presented in greater quantities in the 5 wt % specimen than the 2 wt % one. This introduces a higher stress concentration to the epoxy medium, and therefore results in relatively lower strength observed in the indentation tests.

Micromechanically, stress concentration is higher surrounding a rod-shaped particle as opposed to the concentration surrounding round or elliptical particles, which will lead to a higher stress gradient upon external loading (e.g., compression or tension). Such a stress concentration effect rising from morphological variations from 1 to 5 wt % should have a downgrading effect on relatively large specimens. Further details are provided in Section 3.3.2.

In contrast to the gradual increase seen in the modulus of the 1 wt % case, see Figure 6b, a rapid increase has been observed for several indentations for the 5 wt % case as shown in Figure 10c, and therefore can be inferred as the presence of a higher constraining effect of CNPs in the 5 wt % case. The indentation and SEM results appear to indicate that the CNPs were not very evenly distributed throughout the material in the scale captured by the nanoindentation (0.2 mm × 0.15 mm); however, based on the 3D X-ray CT data presented in Figure 4, the dispersion is reasonably uniform over a region of 2 mm × 2 mm (covering larger than 100 times the area that the SEM and indentation cover), and considered acceptable for further analysis on a structural scale, i.e., tensile and compressive tests.

#### 3.3.2. Tension and Compression Test Data

**Figure 12:**
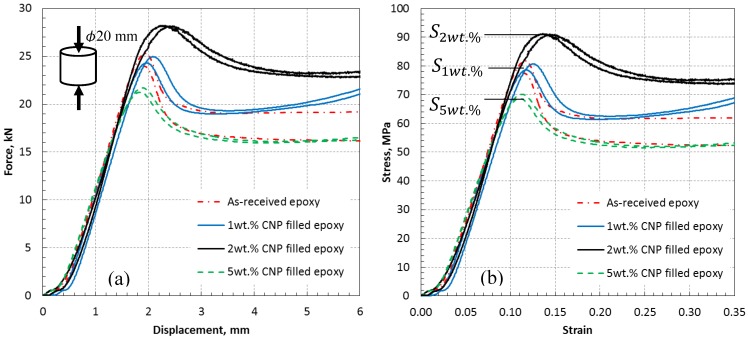
Variation of mechanical response to compression with CNP wt % depicting the maximum threshold at load carrying capacity and maximum stress; (**a**) force versus displacement, (**b**) stress versus strain.

Nanoindentation tests help to realize the local mechanical response of CNPs at the micro-scale, especially surrounding CNPs, in relation to the morphologies that appear in the SEM images. Mechanical testing at the macro-scale using laboratory-scale specimens is required to obtain the properties when numerous CNPs are present. The tension and compression specimens shown in Figure 1 were tested using the uniaxial Tinius testing machine. Both types exhibited a linear elastic behavior until they reach their yield strength point. Young’s modulus was obtained from the slope of the linear elastic part of the strain-stress data. The stress values in the tension specimens did not reach the yield point as they failed prematurely at the grip corners, see Figure 1, due to stress concentration, not in the central region. Compression tests were successfully carried out to the point of the ultimate failure and clearly showed the softening and hardening region. Figure 12 presents compression data in the form of force-displacement and stress-strain curves.

**Figure 13:**
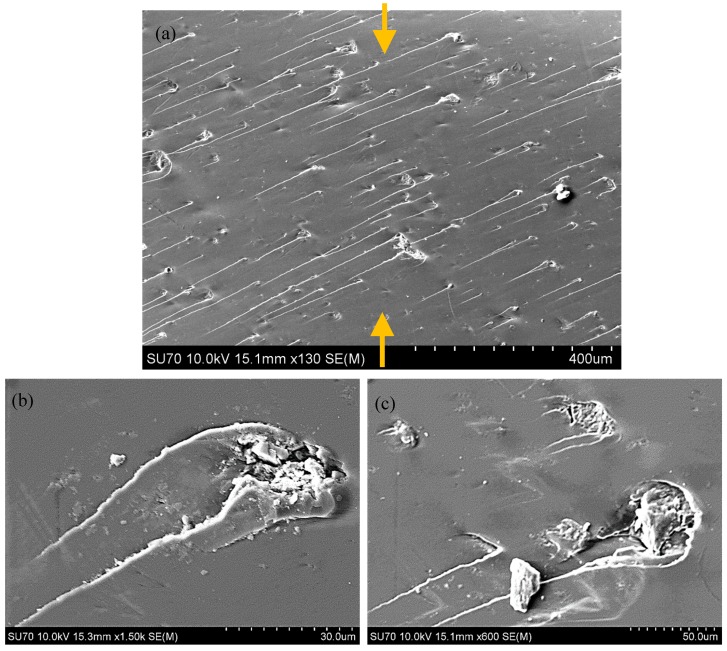
Stress concentration induced microcracks surrounding CNPs in specimens under compression loading; (**a**) microcrack coalesce over a region of 1000 × 700 microns (arrows represent the direction of the compressive load), (**b**,**c**) magnified images of microcracks over regions of 80 × 60 microns.

For the red dotted lines (0 wt %; as-received epoxy), the discrepancy was the highest between the two testing data immediately after the maximum load and stress were reached. This is attributed to the inherent toughness sensitivity (introduction of microcracks) of epoxy (and not yielding or maximum stresses) to variabilities associated with process parameters control, i.e., uncertainties. Data for the as-received and filled epoxies show a slight improvement in the compressive strength when 1 wt % is incorporated (S_(1 wt %) = 79.86 MPa, S_(0 wt %) = 79.84 MPa). An apparent increase is obtained from 2 wt % CNP inclusion (S_(1 wt %) = 91.13 MPa). This increasing trend is reversed when 5 wt % CNP is incorporated (S_(1 wt %) = 69.40 MPa). The reduction for the 5 wt % is consistent with the tensile and compressive modulus (presented in Table 2) and with the indentation data, see Figure 10. The presence of a relatively high quantity of rod fillers should increase the surface interaction between epoxy and CNPs and thus increase the strength by increasing the sectional area of the interaction surfaces. This expected trend is apparent for the 1 and 2 wt % cases. However, it is degraded in the 5 wt % case, most probably due to the shape of the fillers which imposes a stress concentration at the relatively sharp ends of the fillers, and causes high stress gradients transferring to the surrounding epoxy. This phenomenon is apparent in the SEM images from the tested samples, see Figure 13. Arrows in Figure 13 identify the compression loading direction (vertical) which creates a tilted angle with the direction of microcrack coalescence, an indication of brittle fracture behavior normally occurring in a thermosetting polymer matrix. Therefore, a bi-axial positive deformation occurs across the surface perpendicular to the compression direction and in out-of-plane directions, mainly due to the principle of incompressibility in solid bodies. The deformation in the direction of the applied compression is expectedly negative. According to the SEM images, the stress concentration surrounding nanoparticles causes damage initiation in the brittle medium of the epoxy that propagates in the principle shear plane tilted from the loading direction in the epoxy as the weaker medium. This behavior is the same in the presence of all wt %. However, its effect is more influential in the 5 wt % sample as microcracks occurring surrounding the CNPs are denser and require a lower strain energy to coalesce which leads to macro-cracks and therefore, lower strength compared to the 1 and 2 wt % samples.

The tension and compression test data are summarized in Table 2. The modulus and hardness data from nanoindentation tests are also tabulated for comparison. The increasing trend is seen for all properties with an increasing wt % from 0 to 2. The table also shows a degradation trend for all properties from 2 to 5 wt %. Compressive failure strain at which the compressive strength is reached is approximately identical for all specimens (average 11.47%), except for the 2 wt % case where the failure strain slightly increases to 14%.

## 4. Conclusions

The mechanical performance of the CNP filled epoxy was studied in this work. CNP weight percentage was varied between 1% and 5%, and their mechanical properties were derived on a laboratory-scale under tension and compression, and in micro-scale using nanoindentation. A simple embedment procedure was suggested and was applied for all specimens during processing. All data exhibited an increasing trend in the properties with an increasing CNP content to 2 wt %, and a significant degradation for the 5 wt % incorporation. Studies of the morphological change showed densely dispersed rod CNPs in the 5 wt % specimen, which is believed to be the dominant mechanism for mechanical degradation in the specimen. The effect of loading on re-agglomeration was not studied in this paper. However, it is well-known that the mixing time has a significant effect on the final architecture of the nanoparticles in the cured epoxy. The results for the 2 wt % CNP were promising and showed considerable improvements in all the basic properties. The significant increase in the compressive failure strain for this case (21%) also suggests improvements in energy absorption behavior of the epoxy in the presence of 2 wt % CNP. During the examinations, the materials and embedment procedure were constant. Therefore, adhesion properties can be assumed to be identical for all specimens. Thus, the noted improvement with the 2 wt % CNP can be assumed to be due to the smooth stress transfer between the nanoparticle and epoxy constituents.

## Figures and Tables

**Table 1:** Carbon nanoparticles (CNP) embedment procedure.

Stage	Time (*hh:mm)	Stage Description
1	00:00	One hour stirring epoxy (13 mL), DDSA (8 mL), and MNA (7 mL) at RT
2	01:00	Increase the epoxy mixture temperature to 100 °C Add CNPs Stir the CNP-epoxy mixture at 100 °C for 20 min
3	01:20	Turn off heating Continue stirring for 2 h at **RT under vacuum condition
4	03:20	Add curing agent DMP-30 (2 mL = 15 drops) at RT Stir for 21 h under vacuum conditions using a Schlenk line
5	15:20	Inject the mixture into the mold
6	16:00	Cure the molded mixture using vacuum assisted oven at 80 °C
7	28:00	Take out the mold and let cool down for an hour
8	29:00	Open the mold and take out the specimen

*hh:hour, mm: minute, **DDSA: Dodecenylsuccinic anhydride, MNA: Methyl nadic anhydride, ***RT: room temperature.

**Table 2:** Variation of mechanical test data with increasing CNP wt %.

CNP wt %	Tensile Modulus, GPa	Compressive Modulus, GPa	Nanoindentation Compressive Modulus, GPa	Compressive Strength, MPa	Nanoindention Hardness, MPa	Failure Strain, %
**0**	1.18	0.99	3.3	79.84	160	11.55
**1**	1.21 ⬆ (+3%)	0.99 ⬆ (<1%)	3.4 ⬆ (+3%)	79.86 ⬆ (<1%)	180 ⬆ (+13%)	12.15 ⬆ (+5%)
**2**	1.24 ⬆ (+5%)	1.03 ⬆ (+4%)	3.6 ⬆ (+9%)	91.13 ⬆ (+14%)	180 ⬆ (+13%)	14.00 ⬆ (+21%)
**5**	0.83 ⬇ (−30%)	0.84 ⬇ (−15%)	3.4 ⬆ (+3%)	69.40 ⬇ (−13%)	120 ⬇ (−25%)	11.10 ⬇ (−4%)
